# All-Dielectric Phase-Gradient Metasurface Performing High-Efficiency Anomalous Transmission in the Near-Infrared Region

**DOI:** 10.1186/s11671-021-03616-w

**Published:** 2021-10-20

**Authors:** Tiesheng Wu, Zhihui Liu, Yiping Wang, Huixian Zhang, Zuning Yang, Weiping Cao, Dan Yang

**Affiliations:** 1 Key Laboratory of Optoelectronic Devices and Systems of Ministry of Education and Guangdong Province, College of Optoelectronic Engineering, Shenzhen University, Shenzhen, 518060 China; 2 Guangxi Key Laboratory of Wireless Broadband Communication and Signal Processing, School of Information and Communication, Guilin University of Electronic Technology, Guilin, 541004 China; 3 Guangdong and Hong Kong Joint Research Centre for Optical Fiber Sensors, College of Optoelectronic Engineering, Shenzhen University, Shenzhen, 518060 China; 4 Science and Technology on Communication Networks Laboratory, Shijiazhuang, 050000 China

**Keywords:** Metasurface, Phase gradient, Anomalous transmission, All-dielectric

## Abstract

We propose and numerically demonstrate a phase-gradient metasurface with high anomalous transmission efficiency and a large anomalous refraction angle that consists of discontinuous regular hexagonal nanorods supported by a silica substrate. The metasurface achieves high anomalous transmission efficiency and a full 2$\pi$ phase shift for the wavelength range of 1400–1600 nm. At a central wavelength of approximately 1529 nm, the total transmission efficiency reaches 96.5%, and the desired anomalous transmission efficiency reaches 96.2%, with an anomalous refraction angle as large as 30.64. With the adjustment of the period and the number of nanorods per periodic interval, the anomalous transmission efficiency exceeds 69.6% for a large anomalous refraction angle of 68.58. The superior performance of the proposed design may pave the way for its application in optical wavefront control devices.

## Introduction

In recent years, phase-gradient metasurfaces have attracted increasing attention, because they have offered a new pathway for advanced wavefront engineering [1–7]. Compared to conventional wavefront control devices, phase-gradient metasurfaces are much more flexible, making it possible to modulate the amplitude and phase of light [8–11]. Additionally, as a kind of two-dimensional metamaterial, they are easier to apply in the field of photonic integration systems. Since Yu et al. proposed a V-shaped antenna array as a phase-gradient metasurface and explained the concept of the generalized refraction law in detail [12], various phase-gradient metasurfaces based on discrete nanoantenna arrays have been proposed and investigated [2–13]. For instance, Liu et al. introduced a gold grating into a V-shaped gold antenna array, increasing the anomalous transmission efficiency to 15 times that without a gold grating [14]. Phase-gradient metasurfaces have been used in many fields, and their applications include deflectors [8, 15–17], directional surface wave couplers [18, 19], holographic devices [20–22], and vortex beam generators [23–25]. Although the application prospects of metal-based metasurfaces have been verified in many fields, the performance of metasurfaces is usually limited by the very high intrinsic ohmic losses of the metal materials [26, 27]. Because dielectric materials have no intrinsic ohmic loss, people have tried to replace metal materials with dielectric materials in the design of all-dielectric metasurfaces with high performance [28, 29].

More recently, the common challenge in the use of all-dielectric phase-gradient metasurfaces has been the difficulty in achieving high anomalous transmission efficiency with a large anomalous refraction angle. To solve this problem, Zhou et al. designed a metasurface consisting of a gradient array of circular silicon nanorods arranged on a quartz substrate, achieving an anomalous transmission efficiency of 71% with an anomalous refraction angle of 19.27 [6]. Yang et al. designed an all-dielectric metasurface based on silicon nanoantennas for high-efficiency anomalous transmission, the anomalous transmission efficiency of which reached 80.5% with an anomalous refraction angle of 29.62 [30]. In 2019, facilitated by a cross-shaped structure, the anomalous transmission efficiency of an all-dielectric metasurface reached 83.5% with an anomalous refraction angle of 30 [31]. In particular, David Sell et al. proposed and experimentally investigated a periodic dielectric metasurface. In this work, the authors were able to numerically and experimentally observe anomalous refraction with high efficiency (>90%) for outgoing angles up to 50 [32]. In addition, some researchers have used the advantages of hyperbolic metamaterials with broadband and high birefringence to achieve high transmission efficiency [33, 34].

In this work, our goal is to design an all-dielectric metasurface to simultaneously obtain high anomalous transmission efficiency and expand the anomalous refraction angle. The proposed metasurface consists of discontinuous regular hexagonal silicon nanorods supported by a silica substrate. We systematically analyze the anomalous transmission efficiency and the anomalous refraction angle of the proposed structure by using the finite-difference time-domain (FDTD) method. The results show that at a central wavelength of 1529 nm, the total transmission efficiency of the dielectric metasurface can reach 96.5%; moreover, the section of the desired anomalous transmission efficiency can be as high as 96.2% with an anomalous refraction angle of 30.64. The anomalous refraction angle can be enlarged by adjusting the number of elements per periodic interval and the period. We numerically demonstrate an anomalous refraction angle that reaches 68.58 with an anomalous transmission efficiency as high as 69.7% for a central wavelength of 1536 nm. It is believed that the proposed all-dielectric metasurface will play a vital role in advanced wavefront engineering.

## Design and Methods

**Fig. 1:**
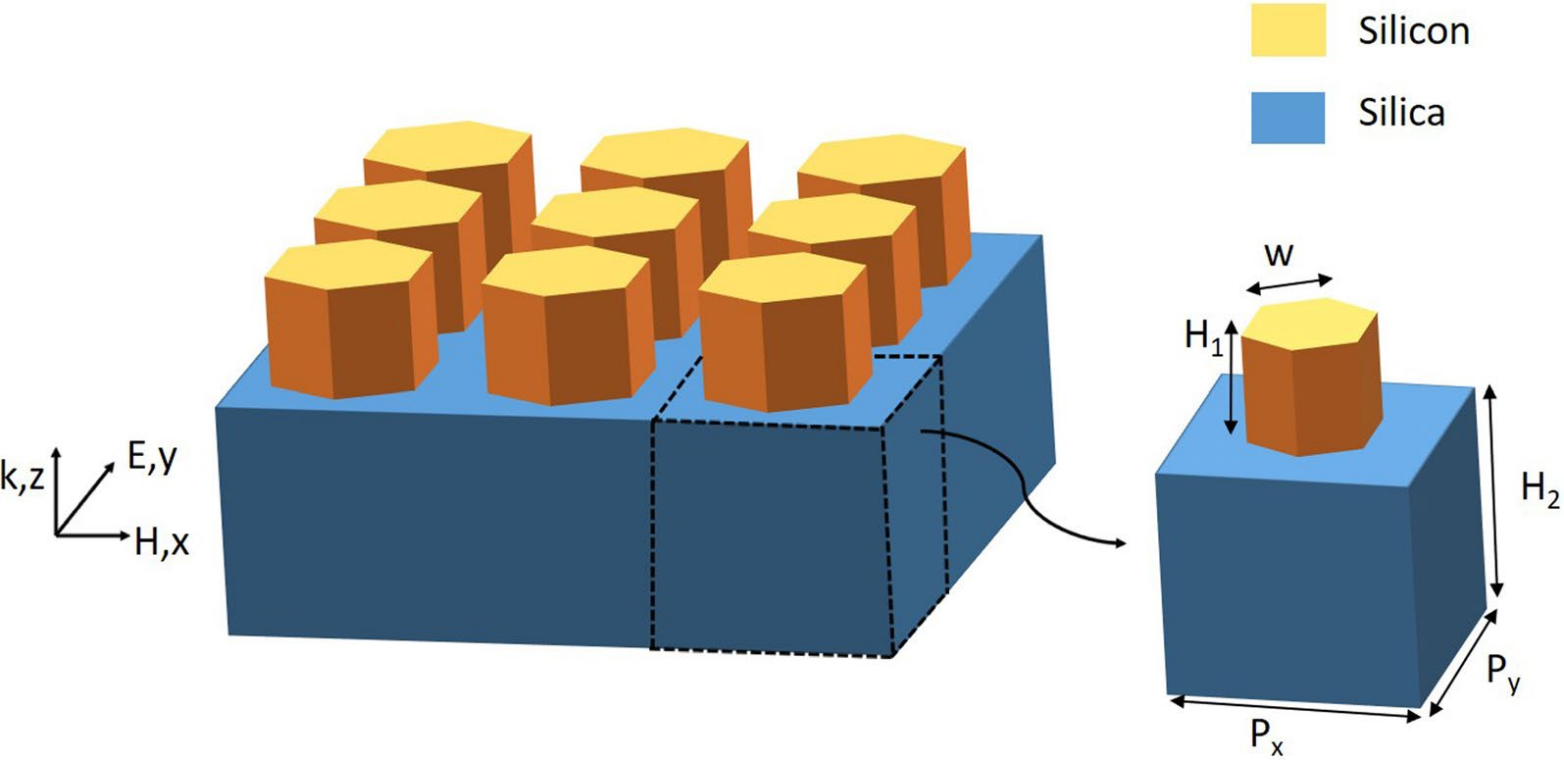
Schematic of a simple array structure composed of regular hexagonal silicon nanorods on a silica substrate

For a phase-gradient metasurface, the geometrical morphology and parameters greatly influence the device performance. As shown in Fig. 1, we first investigate a simple array structure composed of regular hexagonal nanorods based on a silica substrate. The transmission efficiency and phase distributions of the simple array structure are analyzed by using the FDTD method. In the simulation, the *x*- and *y*-directions are set as periodic boundary conditions, and the *z*-direction is set as perfectly matched layers. We set a normal transverse electric (TE) wave to be incident on the bottom. The electric field direction of the incident light is along the *y*-direction, and the wavelength range is 1400–1600 nm. In the numerical analysis, the refractive indexes of silicon and silica are taken from the data proposed by Palik [35]. Experimentally, to fabricate a half-unlimited silica substrate, an etching process must be performed. We also need to deposit a 1200 nm silicon film on top of the silica substrate by using the low-pressure chemical vapor deposition (LPCVD) method. The silicon film is spin-coated with ZEP520A photoresist, and then a thin layer of Cr is deposited as the resist. Hexagonal dielectric nanorods can be obtained by electron beam lithography (EBL). Finally, remover 1165 and $O_2$ plasma are used to remove the photoresist, yielding the designed all-dielectric phase-gradient metasurface [4, 6]. However, the cross section of regular hexagonal nanorods may resemble a circle due to proximity effects in practical experimental fabrication. To solve this problem, we can adjust the proximity effect correction (PEC) and the dose of EBL according to the sample morphology. By adjusting the scheme, we believe that we can eventually obtain precisely manufactured regular hexagonal metasurfaces.

Different from the ideal boundary, when light propagates through the metasurface, optical properties, such as the polarization state, phase, and wavefront, change substantially. We cannot explain these phenomena with the classical Snell’s law in geometric optics when electromagnetic waves propagate through these interfaces, giving rise to a universal generalized Snell’s law [8–12]. Based on the generalized Snell’s law, anomalous reflection or refraction at the interface of two media occurs because of the horizontal phase distribution. We can express the two types of refraction as

1 $$\begin{aligned} \begin{aligned} n_r\sin \theta _r-n_i\sin \theta _i = \frac{\lambda _0}{2\pi }\frac{{\hbox {d}}\phi }{{\text {d}}x} \end{aligned} \end{aligned}$$

where $\theta _r$ represents the refraction angle or anomalous refraction angle and $\theta _i$ represents the incident angle. The refractive index $n_r$ usually refers to the refractive index of air, which has a magnitude of 1. In contrast, $n_i$ refers to the refractive index of the metasurface material, $\lambda _0$ is the operating wavelength in free space, and d$\phi$/${\text {d}}x$ is the phase gradient. The phase-gradient metasurface needs to achieve a complete near-linear $2\pi$ phase shift over a large period to control the anomalous transmission; thus, the phase gradient is

2 $$\begin{aligned} \begin{aligned} \frac{{\hbox {d}}\phi }{{\text {d}}x} = \frac{2\pi }{P_x} \end{aligned} \end{aligned}$$

where $P_x$ is the period of the proposed metasurface along the *x*-axis. In this work, we consider only the normal light incident on the interface; thus, $\theta _i$ is 0, and the equation can be further simplified as

3 $$\begin{aligned} \begin{aligned} sin\theta _r = \frac{\lambda _0}{2\pi }\frac{{\hbox {d}}\phi }{{\text {d}}x} =\frac{\lambda _0}{P_x} \end{aligned} \end{aligned}$$

Phase-gradient metasurfaces exhibit not only low-order anomalous transmission but also high-order anomalous transmission. To determine the high-order anomalous refraction angle, we introduce the grating equation to modify the generalized Snell’s law [36–38]. The modified generalized Snell’s law is

4 $$\begin{aligned} \begin{aligned} \sin\theta _r = m\frac{\lambda _0}{P_x}+\frac{\lambda _0}{P_x} =(m+1)\frac{\lambda _0}{P_x} \end{aligned} \end{aligned}$$

where *m* represents the traditional diffraction order. Electromagnetic wave shifts from the position of the original zeroth order to the position of the first order can be used to determine the anomalous refraction angle. In addition, the period and the operating wavelength determine the total number of diffraction orders. The ratio of $\lambda _0$ to $P_x$ influences the desired value of m. When $\lambda _0$/$P_x$ is greater than 0.5, *m* can take only a value of 0, in which case only three diffraction orders can be obtained: 0, −1, and 1. However, when $\lambda _0$/$P_x$ is less than 0.5, *m* can take a value of either 0 or 1, in which case five diffraction orders can be obtained: $-2, -1, 0, 1$, and 2. In the following discussion, this theory is proven by our calculated results.

To explain the characteristics of the proposed structure, we mainly calculate the efficiency and refraction angle for anomalous transmission. The total transmission efficiency and the anomalous transmission efficiency are defined as

5 $$\begin{aligned} T= I_{\mathrm{out}}/I_{\mathrm{in}} \end{aligned}$$

6 $$\begin{aligned} \eta= I_r/I_{in} \end{aligned}$$

where $I_{\mathrm{in}}$ is the input intensity, $I_{\mathrm{out}}$ is the total transmission intensity, and $I_r$ is the transmitted intensity along the anomalous refraction angle.

**Fig. 2:**
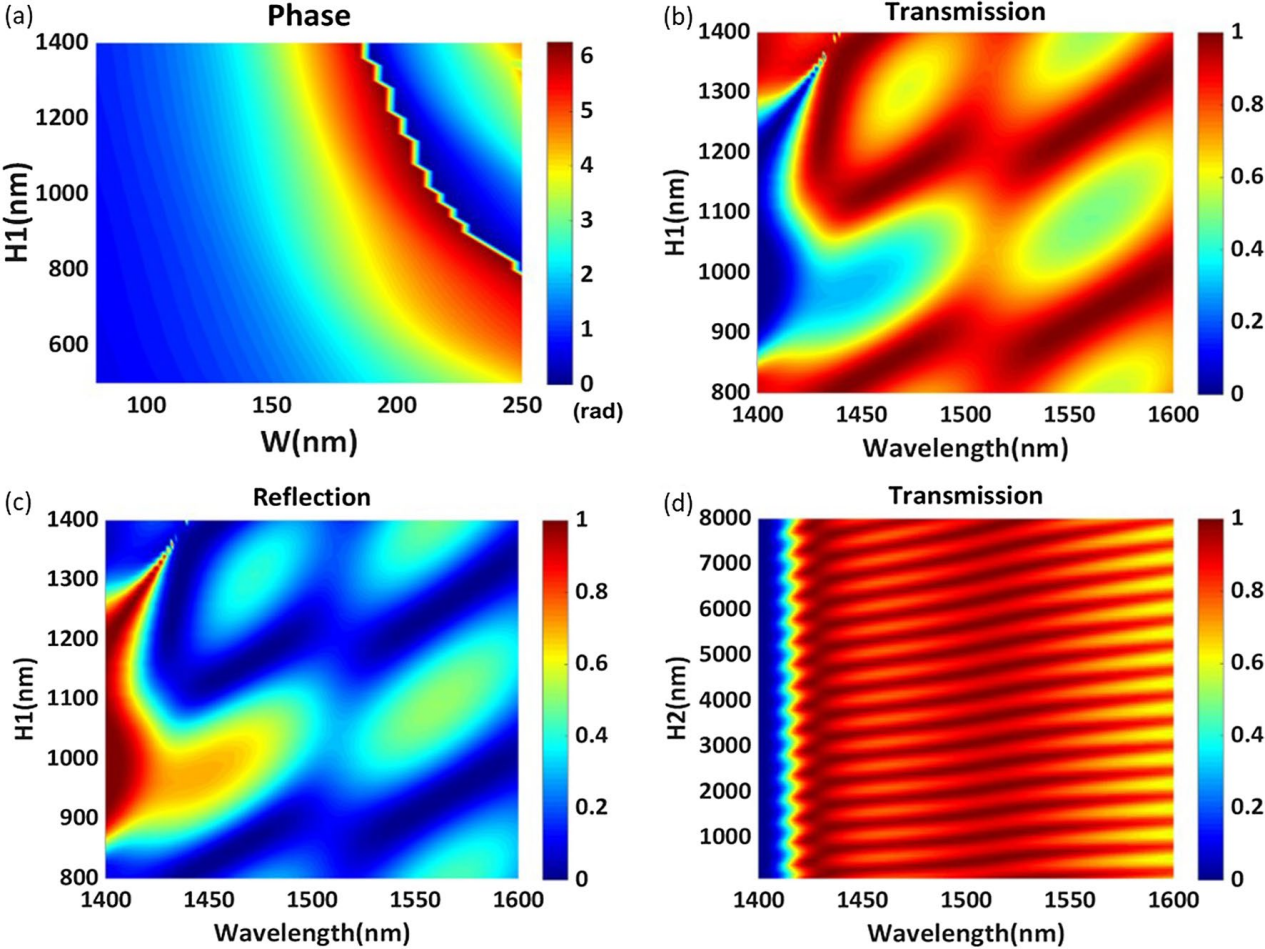
**a** Phase of the periodic regular hexagonal nanorods for different structural parameters $H_1$ and *w* at a wavelength of 1529 nm. **b** The transmission efficiency and **c** the reflection efficiency of the periodic structure for different thicknesses $H_1$ in the wavelength range of 1400–1600 nm. **d** Transmission efficiency of the periodic structure for different thicknesses $H_2$ in the wavelength range of 1400–1600 nm

For the proposed structure, we hope to achieve a complete 2$\pi$ phase shift by adjusting the height $H_1$ and the side length of the regular hexagon *w*. We set the period *P* to 500 nm, and set the substrate thickness $H_2$ to 7050 nm. Since the substrate thickness $H_2$ is greater than $4\lambda$, we can consider the substrate to be a half-unlimited substrate. The phase variations with the change in $H_1$ and *w* at a wavelength of 1529 nm are shown in Fig. 2a. It is clear that the phase of transmitted light varies with the side length of the regular hexagon *w*, but only when the height $H_1$ is greater than 800 nm can this structure realize a full 2$\pi$ phase shift. High transmission efficiency is another factor that needs to be considered when designing phase-gradient metasurfaces. Figure 2b, c shows the changes in the transmission efficiency and reflection efficiency with the wavelength for different heights $H_1$ of the periodic nanorods, shown in Fig. 1. The structural parameter *w* is set to 160 nm. As shown in Fig. 2b, the wavelength of the peak transmission efficiency redshifts with increasing nanorod height. Obviously, the height of the nanorods has a notable effect on the transmission efficiency and reflection efficiency. Here, to obtain high transmission efficiency, the height $H_1$ is set to 1200 nm. At this value, the highest transmission efficiency of the simple homogeneous metasurface is as high as 98.70% at a wavelength of 1540 nm. Figure 2d describes the change in transmission efficiency with the wavelength for different heights $H_2$. The transmission efficiency changes periodically with increasing substrate thickness $H_2$.

**Fig. 3:**
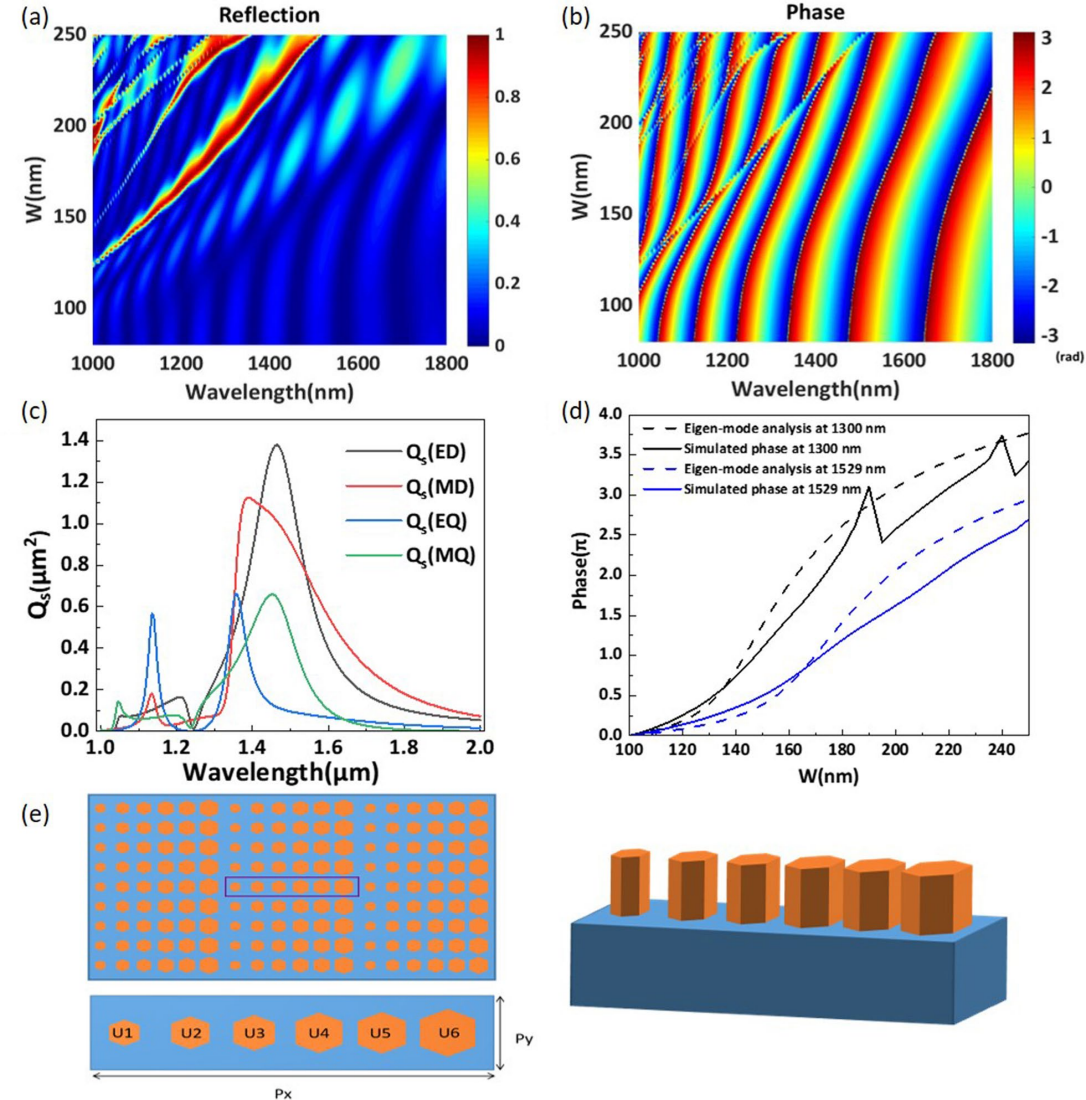
**a** The reflection efficiency and **b** the phase of the periodic regular hexagonal nanorods for different values of *w* in the wavelength range of 1000–1800 nm. **c** Scattering cross section $Q_s$ versus the wavelength of an isolated regular hexagonal silicon nanorod. The contribution of each term to the Mie expansion is shown. **d** Phase profiles obtained via eigenmode analysis and numerical simulations for varying side length *w*. **e** Schematic of the designed phase-gradient metasurface

Figure 3a, b illustrates the variation in the reflection efficiency and phase of the simple array structure by changing the side length of the regular hexagons for the wavelength range of 1000–1600 nm. As shown in Fig. 3a, b, there are many distinguishable resonant peaks in the reflection spectrum. Through the simple array structure, a nearly $\pi$ phase shift can be realized for each resonant wavelength. It is clear that a full $2\pi$ phase shift can be achieved when the side length of the regular hexagon *w* changes from 100 to 220 nm at a wavelength of 1529 nm. To further clarify the mechanism of the $2\pi$ phase shift, we use the electromagnetic multipole expansion (EME) method to calculate the scattering cross sections (SCSs) of an isolated regular hexagonal silicon nanorod [31, 41]. In Fig. 3c, we plot the calculated scattering SCSs of the electric dipole (ED), magnetic dipole (MD), electric quadrupole (EQ) and magnetic quadrupole (MQ) components for *w*=160 nm. Obviously, various Mie resonances, especially dipole resonances, are excited at the operating wavelength. However, there are some deviations between the excitation of the Mie resonances in the isolated particle and that in the periodic particles. There is no abrupt phase change at a wavelength of 1529 nm, which proves that the $2\pi$ phase shift is formed by only one mode. Therefore, the $2\pi$ phase control mechanism at a wavelength of 1529 nm is analyzed by eigenmode analysis [42]. These nanorods can be considered low-quality-factor Fabry–Pérot resonators, and the phase can be modulated by the effective refractive index of the fundamental mode. Thus, the phase can be demonstrated to be

7 $$\begin{aligned} \begin{aligned} \varphi = H_1*n_{\mathrm{eff}}*2\pi /\lambda \end{aligned} \end{aligned}$$

where $H_1$ is the height of these nanorods, $n_{\mathrm{eff}}$ is the effective refractive index of the fundamental mode obtained by eigenmode analysis, and $\lambda$ is the operating wavelength. In Fig. 3d, we plot the phase profiles obtained via eigenmode analysis (dashed line) and numerical simulation (solid line) at wavelengths of 1300 nm and 1529 nm, respectively. As shown in Fig. 3d, there are two abrupt phase reductions in the simulated phase at a wavelength of 1300 nm, corresponding to two kinds of Mie resonances. When *w* changes from 100 to 250 nm, the phase change trends obtained by the two methods are basically the same at a wavelength of 1529 nm. According to the redshift of the reflection peaks in Fig. 3a, when *w* is greater than 250 nm, the Mie resonance is excited at a wavelength of 1529 nm. For the metasurface we propose in this work, since the structural parameters of each element are in the range of 100 to 220 nm, as shown in Table 1, no Mie resonances are excited within this range. Therefore, we can assume that the phase shift is mainly based on the Fabry–Pérot resonance [6, 39, 40, 42]. According to the generalized Snell’s law, anomalous transmission can be achieved if a metasurface has a $2\pi$ phase shift ability. By adjusting the size of the nanorods so that the phase shift is evenly spaced and covers a full $2\pi$ range, we can deflect the beam by dislocating its wavefront. Figure 3e illustrates the schematic diagram of the phase-gradient metasurface. Six silicon nanorods of different sizes with $2\pi /5$ phase intervals are arrayed on a silica substrate to form a complete phase-gradient from 0 to $2\pi$. The purple box represents a complete period, and $P_x$ and $P_y$ are set to 3000 nm and 500 nm, respectively.

**Table 1:** The side lengths of the regular hexagonal nanorods

Unit cell	$U_1(0)$	$U_2(2\pi /5)$	$U_3(4\pi /5)$	$U_4(6\pi /5)$	$U_5(8\pi /5)$	$U_6(2\pi )$
*w* (nm)	113	144	158	179	180	211

**Fig. 4:**
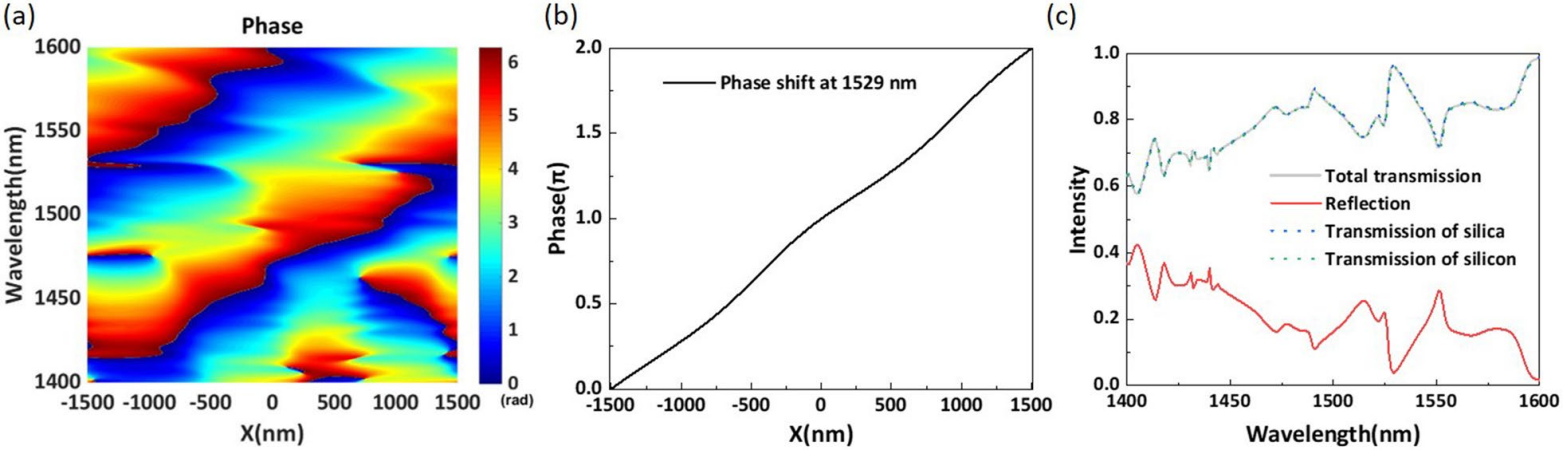
**a** Simulated phase shift of the metasurface along the *x*-direction in a complete period for the wavelength range of 1400–1600 nm. **b** Simulated phase distribution along the *x*-direction at a wavelength of 1529 nm. **c** Simulated intensities of the transmitted and reflected light

## Results and Discussion

Table 1 shows the structural parameters of each element for the proposed structure. We investigate the phase distribution and intensity of the transmission light. To facilitate the analysis, we set the origin of the coordinates as the center of the supercell. We simulate the phase distribution of the transmission light in the wavelength range of 1400–1600 nm. As shown in Fig. 4a, the proposed structure can realize a full $2\pi$ phase shift in the range of 1400–1600 nm. To make this clear, Fig. 4b shows the phase shift curve at a central wavelength of 1529 nm. As depicted in Fig. 4b, the phase shift shows a linear trend and is very smooth. According to the generalized Snell’s law, the better the linearity of the phase shift is, the flatter the equip phase plane of the transmitted light. We simulate the transmittance and the reflectance of the proposed metasurface for the range of 1400–1600 nm, the results of which are shown in Fig. 4c. By observing the curve, we can see that the total transmission remains highly efficient, exceeding 60% over the whole operating wavelength range. At a wavelength of 1529 nm, the total transmission efficiency reaches 96.5% with a reflection efficiency of 3.4%. The sum of the reflectivity of the structure and the transmittance of the silica substrate is 1 in the whole wavelength range. Therefore, we can determine that the reflection mainly occurs at the first interface between the air and the substrate. As shown in Fig. 4c, the differences between the three transmission curves are barely discernible and are caused by absorption of the structure. The absorption rate is much less than 0.1% because the imaginary part of the refractive index of silicon in the near-infrared wavelength range is very small. Thus, the absorption rate is negligible. The transmission efficiency and reflection efficiency exhibit opposite trends to that of the wavelength, and the loss of the structure mainly comes from reflection. It is clear that the proposed phase-gradient metasurface can realize a complete near-linear $2\pi$ phase shift and simultaneously maintain higher transmission efficiency in the range of 1400–1600 nm.

**Fig. 5:**
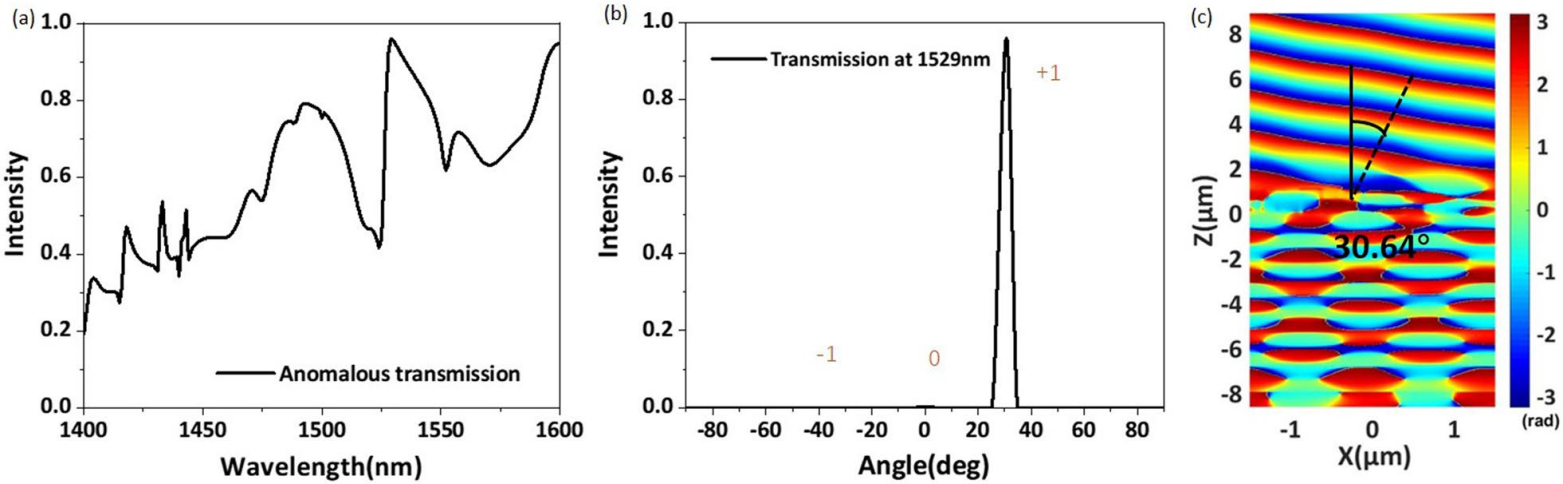
**a** Simulated intensity of the anomalous transmission efficiency. **b** Far-field transmission efficiency for different anomalous refraction angles at a wavelength of 1529 nm. **c** Phase distribution of the metasurface configuration at a wavelength of 1529 nm. The angle in the figure shows the refraction angle of anomalous transmitted light

As shown in Fig. 5a, we also calculate the desired anomalous transmission efficiency of the phase-gradient metasurface over the whole operating wavelength range and normalize it to the energy of the incident light. Comparing Fig. 4c with Fig. 5a, we can see that the trends of the total transmission efficiency and anomalous transmission efficiency with the wavelength are consistent. The results show that the desired anomalous transmission efficiency exceeds 80% in the wavelength ranges of 1527–1545 and 1591–1600 nm. Remarkably, the anomalous transmission efficiency is as high as 96.2% at a wavelength of 1529 nm. Figure 5b shows the relationship between the far-field transmission efficiency and the anomalous refraction angle at a wavelength of 1529 nm. It is clear that the far-field energy of the transmitted light is mainly concentrated at an angle of 30.64, and only weak energy is distributed at the other two angles. For easy observation, Fig. 5c shows the phase distribution of the metasurface configuration at the center wavelength. From Fig. 5c, we can see that the transmitted light is obviously refracted and that the wavefront is relatively flat. By substituting the working wavelength and the period of the structure into Eq. (3), we obtain an anomalous transmission angle $\theta _r$ of 30.642, which is very close to our simulation results. To verify the relationship between the number of diffraction orders and the ratio of the wavelength to the period, we set $\lambda _0$/$P_x$ to the critical value of 0.5 and select five different wavelengths to perform theoretical calculations and FDTD simulations. The results are shown in Table 2. Obviously, the simulation results are very consistent with the calculated results.

**Table 2:** Calculated and simulated angles for orders +1 and +2

$\lambda (nm)$		1495	1499	1504	1508	1510
$\lambda /P$		0.4983	0.4997	0.5013	0.5027	0.5033
Calculation order 1		29.8876	29.9802	30.0860	30.1788	30.2186
Angle (deg) order 2		85.2739	88.0151	–	–	–
Simulation order 1		29.8898	29.9779	30.0883	30.1766	30.2208
Angle (deg) order 2		85.3205	87.9077	–	–	–

According to the calculated and simulated angles for the proposed structure shown in Table 2, when $\lambda _0$/$P_x$ is greater than 0.5, only diffraction order 0 and diffraction order 1 are present, and there is no diffraction order 2. When $\lambda _0$/$P_x$ is less than 0.5, diffraction orders 0, 1, and 2 are obtained in the simulation. This result is in complete agreement with the theoretical analysis described above and thus fully confirms the reliability of the generalized Snell’s law combined with grating theory.

**Fig. 6:**
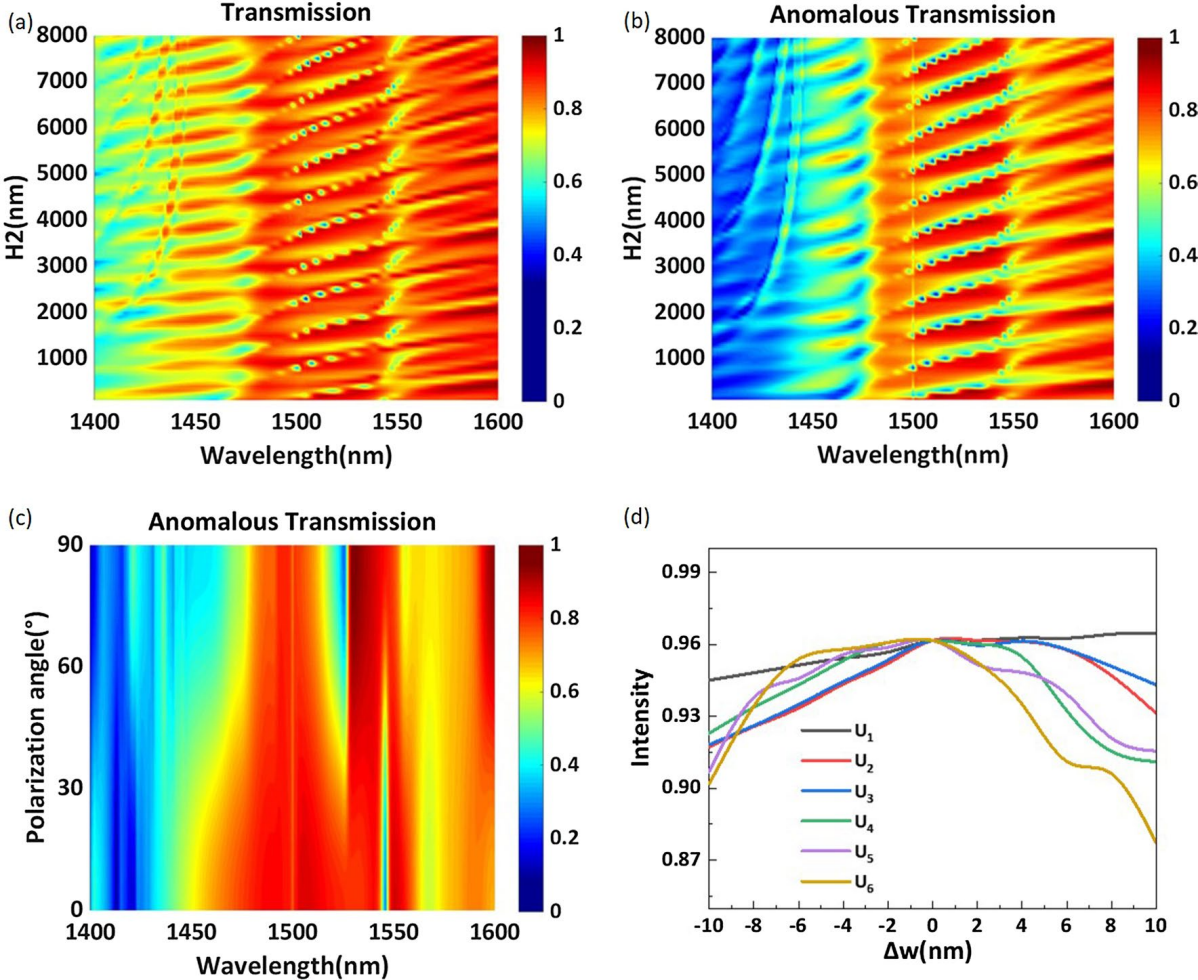
**a** The total transmission efficiency and **b** the anomalous transmission efficiency as a function of the substrate thickness for the wavelength range of 1400–1600 nm. **c** The anomalous transmission efficiency of the proposed structure for different polarization angles in the wavelength range of 1400–1600 nm. **d** Calculated anomalous transmission efficiency at different values of the side length *w*

In Fig. 6a, b, the wavelength range is 1400–1600 nm, and the total transmission efficiency and the anomalous transmission efficiency are plotted as a function of the substrate thickness $H_2$. The transmission efficiency is affected by the substrate thickness, and the peak wavelength is redshifted with increasing thickness. It is obvious that both the total transmission efficiency and the anomalous transmission efficiency change periodically with increasing substrate thickness. To reduce the memory consumption in the computer simulation, the optimized substrate thickness is set as 7050 nm, and the desired anomalous transmission efficiency reaches 96.2% at a wavelength of 1529 nm. We believe that a high anomalous transmission efficiency can be obtained even if the substrate is thick. We also calculate the variation in the anomalous transmission efficiency with the polarization angle of the incident light, as shown in Fig. 6c. At a wavelength of 1529 nm, the anomalous transmission efficiency increases with increasing polarization angle and reaches a maximum when the polarization angle is 90 (*y*-polarization). Considering that the side length *w* of the structure requires precise numerical values and may be difficult to precisely fabricate, we calculate the anomalous transmission efficiency at different values of *w* to test the tolerance of the structure. As shown in Fig. 6d, the tolerance of the structure is obtained by changing the side length *w* based on the structural parameters listed in Table 1. These curves, $U_1$–$U_6$, represent the variation in the anomalous transmission efficiency with the side lengths of the six nanorods per periodic interval. The horizontal axis $\Delta w$ represents the difference between the simulated side length and the side length listed in Table 1. We can see that curve $U_1$ is very flat and that the anomalous transmission efficiency changes by only 2% with the side length within a 20 nm bandwidth. The trends of curves $U_2$,$U_3$, $U_4$, and $U_5$ are basically the same, and greater than 90% anomalous transmission efficiency can be obtained when the side length is within the 20 nm bandwidth. Obviously, changing the side length of $U_6$ has the most notable influence on the performance; nevertheless, $U_6$ still exhibits high anomalous transmission efficiency. When the side length is reduced by 10 nm, the anomalous transmission efficiency remains above 90%. When the side length is increased by 10 nm, the anomalous transmission efficiency is notably affected, but it still exceeds 87%. These results prove that a small error during manufacturing does not substantially affect the metasurface performance.

**Fig. 7:**
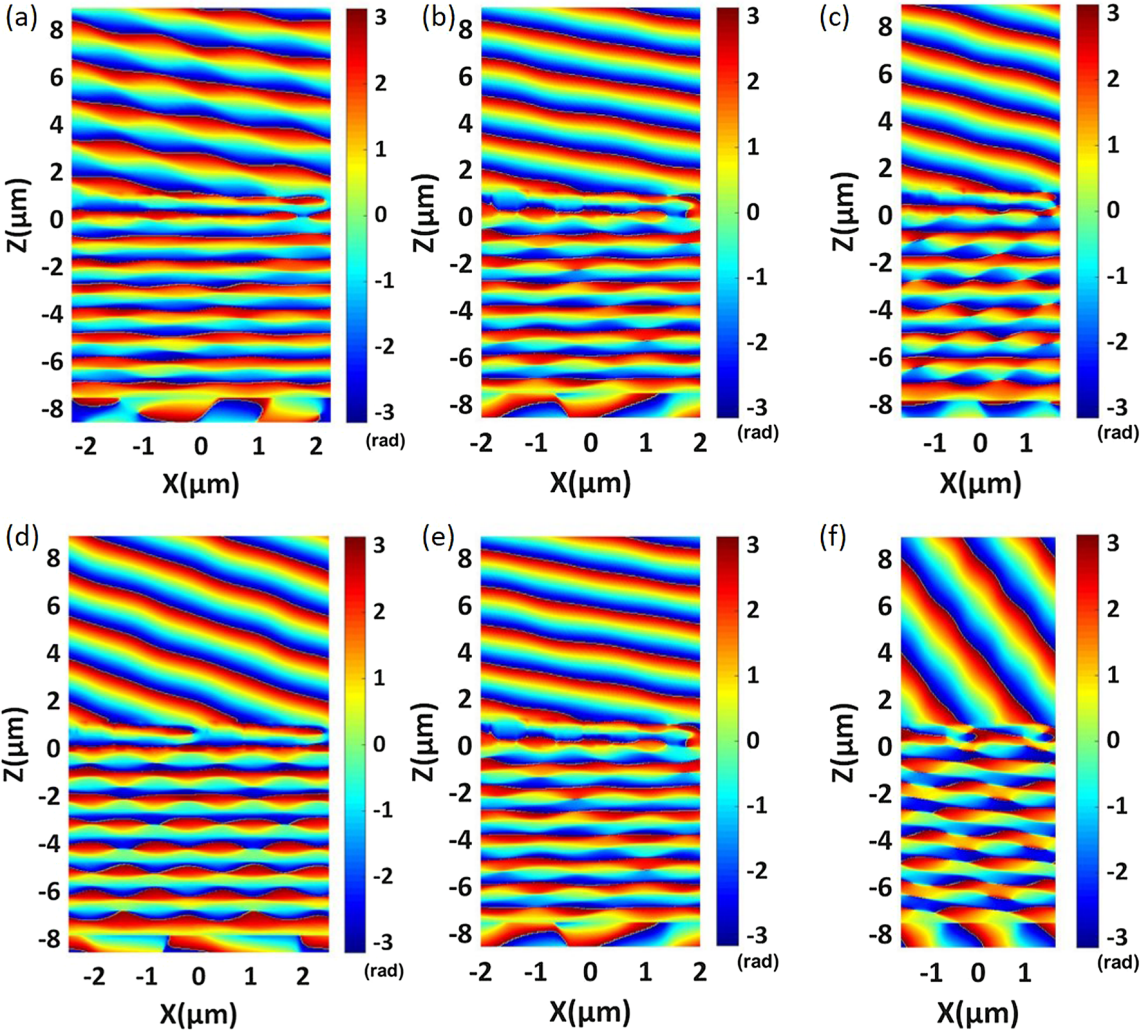
Phase distribution of a phase-gradient metasurface consisting of different element numbers. **a** Nine-element metasurface. **b** Eight-element metasurface. **c** Seven-element metasurface. **d** Five-element metasurface. **e** Four-element metasurface. **f** Three-element metasurface. **d**–**f** depict two periods to better show the anomalous transmission effect. The detailed parameters are shown in Table 3

**Fig. 8:**
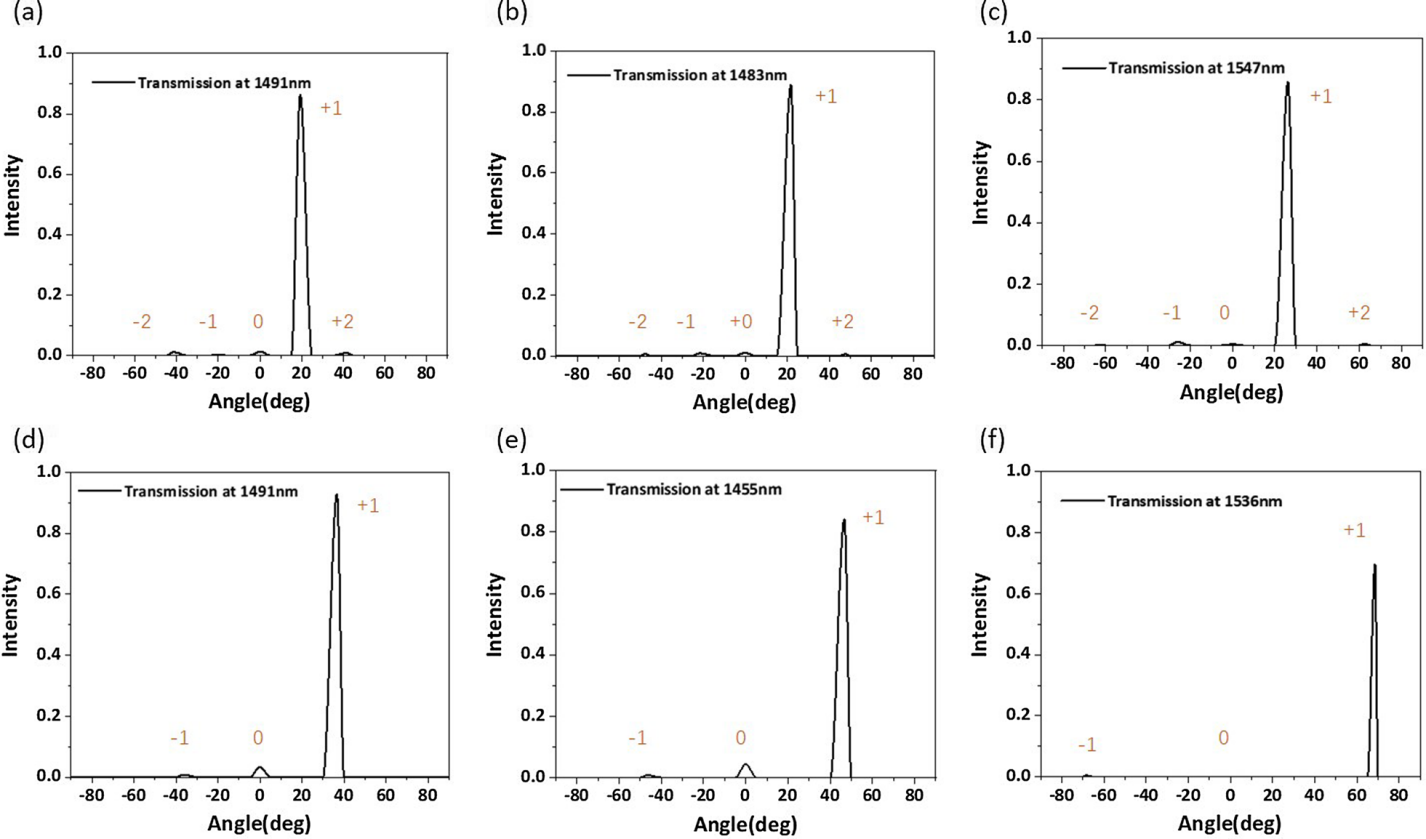
Far-field transmission intensities at different angles of phase-gradient metasurfaces consisting of different element numbers. **a**–**f** represent nine, eight, seven, five, four, and three elements, respectively

It can be seen from Eq. (3) that the diffraction angle of anomalous transmission light is affected by $\lambda _0$/$P_x$; thus, we try to change the magnitude of $P_x$ to obtain different anomalous refraction angles. An effective method for realizing different anomalous refraction angles is to change the number of elements per periodic interval. Therefore, we further design phase-gradient metasurfaces with multiple sets. The elements of the metasurface per periodic interval change from three to nine. We select the working wavelength with the highest anomalous transmission efficiency for each group of metasurfaces and observe the phase distribution of the transmitted light. The simulation results are plotted in Fig. 7a–f. As the number of elements decreases from nine to three, the ratio of $\lambda _0$/$P_x$ increases gradually, and the anomalous transmission angle increases from 19.35 to 68.58. Figure 7a–f shows that phase-gradient metasurfaces with different elements can realize near-linear phase distributions and that the wavefront of the transmitted light is relatively smooth. We carry out far-field analysis of the above configurations and plot the energy distribution of transmitted light along each diffraction angle, as shown in Fig. 8a–f. We can obtain more than 80% anomalous transmission efficiency from 19.35 to 46.68. The structural parameters of each element and detailed numerical results are listed in Table 3. In our optimization process, the side length of the regular hexagon *w* and the period *P* are the main optimization parameters.

**Table 3:** Crucial parameters of phase-gradient metasurfaces consisting of different numbers of elements

*N*	$U_1$	$U_2$	$U_3$	$U_4$	$U_5$	$U_6$	$U_7$	$U_8$	$U_9$	$\eta$ (%)	*P*	$\lambda$	$\theta$
3	0.144	0.183	0.204	–	–	–	–	–	–	69.6	1.65	1.536	68.58°
4	0.080	0.155	0.172	0.203	–	–	–	–	–	84.1	2.0	1.455	46.68°
5	0.111	0.146	0.158	0.171	0.190	–	–	–	–	92.8	2.5	1.491	36.61°
6	0.113	0.144	0.158	0.179	0.180	0.211	–	–	–	96.2	3.0	1.529	30.64°
7	0.120	0.137	0.150	0.170	0.176	0.192	0.214	–	–	85.8	3.5	1.547	26.23°
8	0.089	0.120	0.149	0.150	0.164	0.169	0.185	0.198	–	88.9	4.0	1.483	21.76°
9	0.110	0.140	0.143	0.159	0.166	0.172	0.181	0.197	0.200	86.3	4.5	1.491	19.35°

**Fig. 9:**
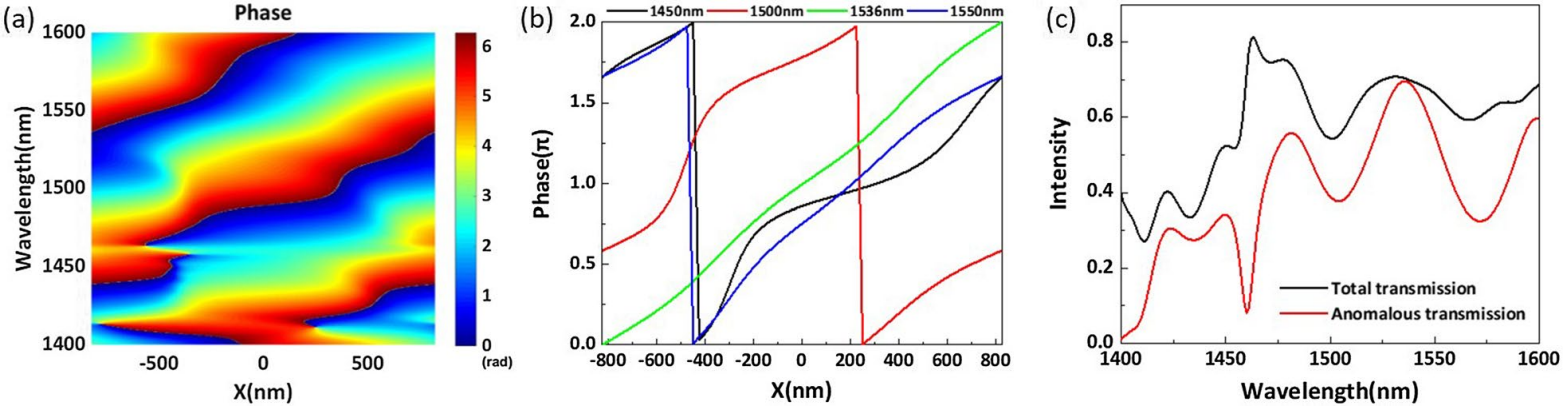
**a** Simulated phase variation of the large angle metasurface along the *x*-direction in a complete period for wavelengths of 1400–1600 nm. **b** Full $2\pi$ phase shift along the *x*-direction of the phase-gradient metasurface for 1450, 1500, 1536, and 1550 nm. **c** The intensity of the total transmission and anomalous transmission

According to the generalized Snell’s Law, to design a larger anomalous refraction angle $\theta _r$, we should increase the ratio of the working wavelength $\lambda$ to the structural period $P_x$. As shown in Fig. 9a, we plot the phase variation of the transmitted light along the *x*-direction for wavelengths of 1400–1600 nm. For clarity, we select four wavelength points, i.e., 1450 nm, 1500 nm, the central working wavelength 1536 nm, and 1550 nm, to plot the phase shift curves shown in Fig. 9b. It is clear that the all-dielectric metasurface can realize a full $2\pi$ phase shift for the wavelength points. From Fig. 9b, we can see that the phase variation shows a linear trend along the *x*-direction. We calculate the total transmission efficiency and the desired anomalous transmission efficiency of the structure in the working band, the results of which are shown in Fig. 9c. It can be observed that the total transmission efficiency is lower than before. However, at the operating wavelength of 1536 nm, the anomalous transmission efficiency can reach 69.6% with an anomalous refraction angle of 68.58. The phase distribution of transmitted light and the energy distributions at different anomalous refraction angles are shown in Figs. 7f and 8f, respectively. From the electric field distribution, we can clearly see that the equilateral phase plane of the transmitted light is very flat. The transmitted light emits very little energy at 0 and $-68.58$, and the majority of transmitted light is concentrated at 68.58. The anomalous transmission performance of the all-dielectric phase-gradient metasurface designed by us is better than that of most of the metasurface structures proposed before, and the anomalous transmission efficiency can reach more than 60% within the range of anomalous refraction angles from 0 to 70. Based on the above analysis, an anomalous refraction angle of approximately 30 is the most reasonable. At this anomalous refraction angle, the highest anomalous transmission efficiency can be achieved, and the anomalous refraction angle can be guaranteed to be large enough.

## Conclusions

In summary, we designed and numerically investigated an all-dielectric phase-gradient metasurface to achieve high-efficiency anomalous transmission in the near-infrared region. The metasurface consists of regular hexagonal silicon nanorods arranged on a silica substrate. The FDTD method was used to calculate the transmission efficiency and anomalous refraction angle of the transmitted light. The results show that the metasurface can realize a complete $2\pi$ phase shift in the wavelength range of 1400–1600 nm. At a center wavelength of 1529 nm, the desired anomalous transmission efficiency reached 96.2% with an anomalous refraction angle of 30.64. Furthermore, the anomalous transmission efficiency exceeded 80% in the range of 1527–1545 nm, which means that our design is more flexible. We also designed multiple sets of phase-gradient metasurfaces by changing the number of elements per periodic interval and adjusting the period of the metasurface. The optimized results show that we can modulate the anomalous refraction angle in the range of 19.35-68.58. When the anomalous refraction angle is less than 46.68, more than 80% of the anomalous transmission efficiency can be obtained. Such an all-dielectric metasurface will be easy to apply to integrated optical devices.

## Data Availability

The datasets generated and analyzed during the current study are available from the corresponding author on reasonable request.

## Abbreviations

FDTD: Finite difference time domain
TE: Transverse electric
LPCVD: Low-pressure chemical vapor deposition
EBL: Electron beam lithography
PEC: Proximity effect correction
EME: Electromagnetic multipole expansion
SCSs: Scattering cross sections
ED: Electric dipole
MD: Magnetic dipole
EQ: Electric quadrupole
MQ: Magnetic quadrupole
